# Value of the urea/creatinine index in isolated urine to estimate severe protein hypercatabolism in ventilated patients

**DOI:** 10.5935/0103-507X.20200087

**Published:** 2020

**Authors:** Dino Moretti, Melisa D. Ré, Nicolás Sebastián Rocchetti, Daniel H. Bagilet, Claudio Jesús Settecase, Martin G. Buncuga, Marta B. Quaglino

**Affiliations:** 1 Universidad Nacional de Rosario - Santa Fe, Argentina.; 2 Intensive Care Unit, Hospital “Eva Perón” - Santa Fe, Argentina.

**Keywords:** Nutritional assessment, Critical illness, Inflammation, Proteins/metabolism, Nutritional support, Evaluación nutricional, Enfermedad crítica, Inflamación, Proteínas/metabolismo, Soporte nutricional

## Abstract

**Objective:**

To study the ability of the urea/creatinine index to identify severe protein catabolism from the isolated urine of critically ventilated patients.

**Methods:**

This was a prospective, observational study. It included 52 patients without kidney failure. Variables: total urinary nitrogen estimated from the urea in 24-hour urine on the second (T1) and fourth days (T2) and urea/creatinine index in isolated urine before 24-hour urine collection.

**Results:**

Severe protein hypercatabolism (estimated total urinary nitrogen > 15g) was present in 14 patients (26.9%) at T1 and in 29 (55.7%) at T2. Eighty-four percent of patients had low nutritional risk by the Nutrition Risk in the Critically Ill score. At T1, the Pearson correlation between the estimated total urinary nitrogen and the urea/creatinine index was 0.272 (p = 0.051), and at T2 it was 0.276 (p = 0.048). The urea/creatinine index at T2 had a tendency to better discriminate severe protein hypercatabolism than Acute Physiology and Chronic Health Evaluation II and Nutrition Risk in the Critically Ill (AUC 0.741 versus 0.669 and 0.656, 95%CI: 0.602 - 0.880; 0.519 - 0.818 and 0.506 - 0.806, respectively). The optimal cutoff value of the urea/creatinine index for the diagnosis of severe protein hypercatabolism was 16.15, with a sensitivity of 79.31% (95%CI: 59.74 - 91.29), specificity of 60.87% (95%CI: 38.78 - 79.53), positive predictive value 71.88% (95%CI: 53.02 - 85.60), negative predictive value 70.0% (95%CI: 45.67 - 87.18), LR (+) 2.03 (95%CI: 1.18 - 3.49), and LR (-) 0.34 (95%CI: 0.16 - 0.74).

**Conclusion:**

The urea/creatinine index measured on the fourth day has a certain ability to estimate severe protein hypercatabolism (as defined by estimated total urinary nitrogen) but does not replace total urinary nitrogen in critically ventilated patients without kidney failure. Due to its reasonable sensitivity, it could be used as a screen to identify which patients to take a 24-hour urine sample from.

## Abstract (es)

**Objetivo:**

Estudiar la capacidad discriminativa de hipercatabolismo proteico grave del índice urea/creatinina en orina aislada en pacientes críticos ventilados.

**Metodos:**

Estudio prospectivo, observacional. Incluyó 52 pacientes sin insuficiencia renal. Variables: nitrógeno urinario total estimado a partir de la urea en orina de 24 horas al segundo (T1) y cuarto día (T2) e índice urea/creatinina en orina aislada previo a la recolección de orina de 24 horas.

**Resultados:**

Presentaron hipercatabolismo proteico grave (nitrógeno urinario total estimado > 15g) 14 pacientes (26,9%) en T1 y 29 (55,7%) en T2. El 84% de los pacientes presentaron bajo riesgo nutricional por la escala *Nutrition Risk in the Critically Ill*. En el segundo día, la correlación de Pearson del nitrógeno urinario total estimado con el índice urea/creatinina fue: 0,272 (p = 0,051) y en el cuarto día: 0,276 (p = 0,048). El índice urea/creatinina al cuarto día, tuvo una tendencia a mayor discriminación del hipercatabolismo proteico grave que el *Acute Physiology and Chronic Health Evaluation II* y *Nutrition Risk in the Critically Ill* (AUC 0,741 versus 0,669 y 0,656, IC95%: 0,602 - 0,880; 0,519 - 0,818 y 0,506 - 0,806 respectivamente). El valor de corte optimo del índice urea/creatinina para diagnóstico de hipercatabolismo proteico grave fue de 16,15 con una sensibilidad de 79,31% (IC95%: 59,74 - 91,29), especificidad de 60,87% (IC95%: 38,78 - 79,53), valor predictivo positivo 71,88% (IC95%: 53,02 - 85,60), valor predictivo negativo 70,0% (IC95%: 45,67 - 87,18), LR (+) 2,03 (IC95%: 1,18 - 3,49) y LR (-) 0,34 (IC95%: 0,16 - 0,74).

**Conclusión:**

El índice urea/creatinina realizado al cuarto día tiene un discreto valor para estimar el hipercatabolismo proteico grave por nitrógeno urinario total y no reemplaza al mismo en pacientes críticos ventilados sin falla renal. Por su razonable sensibilidad podría ser utilizado como cribado para identificar a quien tomar la muestra de orina de 24 horas.

## INTRODUCTION

Protein hypercatabolism (PHC) is recognized as one of the main metabolic alterations of critically ill patients. The increased loss of body protein, usually evidenced by a negative nitrogen balance, implies a decrease in lean mass and an increase in morbidity and mortality.^(1)^ Preserving the quality and function of skeletal muscle are key objectives for maximizing the quality of life and the long-term outcome of patients who survive a critical illness.^(2)^

The 2016 guide on nutritional support in the critically ill patient of the American Society for Parenteral and Enteral Nutrition - Society of Critical Care Medicine (A.S.P.E.N. - SCCM) suggests the use of the Nutrition Risk In the Critically Ill (NUTRIC) and Nutritional Risk Screening 2002 (NRS 2002) scales for the initial nutritional assessment for deciding on the intensity of nutritional therapy, without considering the measurement of PHC.^(3)^

One of the recognized limitations of these scales is precisely that they do not include a variable that quantifies PHC.^(4)^ Our working group showed that the estimators of the metabolic stress response included in NUTRIC do not reflect protein catabolism in the critically ill patient; therefore, this scale should not replace the objective determination of protein catabolism.^(5)^

In this sense, nitrogen balance has limitations in patients with short stays in the intensive care unit (ICU).^(6)^ The known disadvantages of assessing the PHC based on the estimated total urinary nitrogen (TUNes) from the urine urea nitrogen of a 24-hour urine sample (collection of fallible and cumbersome samples, variable and delayed results) constitute the main barrier to its implementation in daily clinical practice; however, it is one of the few tools available in most healthcare centers.^(7-12)^

Creatinine is a component of urinary nitrogen. Its urinary excretion has been correlated with muscle mass and has been used to index nitrogen losses in relation to body composition or even as a nutritional marker.^(7)^ The urea/creatinine index (U/CI) in isolated urine has been proposed as a simple and rapid alternative method to estimate urinary nitrogen excretion. The correlation between U/CI in isolated and 24-hour urine has been shown, as has the correlation between TUNes and TUN derived from the U/CI in isolated urine.^(13-15)^ A study in surgical and stroke patients admitted to the general ward demonstrated the usefulness of U/CI in isolated urine as a tool to measure changes in protein catabolism.^(16)^

Within a solid and coherent physiopathological framework for understanding nutrition in critically ill patients, where malnutrition is closely related to the underlying inflammatory state and the depletion of body protein is central, having a simple, dynamic, and low-cost method of estimating severe PHC (sPHC) is a priority. The objective of this study is to test the hypothesis that U/CI in isolated urine is useful for this purpose.

## METHODS

This study is a follow-up analysis of a prospective and observational study conducted between January 1 and June 30, 2016 in the high-complexity, multipurpose ICU of the *Hospital Escuela “Eva Perón”*.^(5)^ This is a university hospital with 137 beds available for the care of adult patients with acute pathology, of which 14 belong to the ICU.

Patients of both sexes, aged 18 years or older, hospitalized for at least 72 hours in the ICU, and receiving assisted mechanical ventilation (AMV) since admission were included. Patients with anuria, renal insufficiency (acute or chronic), renal replacement therapy, or incomplete data were excluded from the study.

The NUTRIC score contains the following variables: age, Acute Physiology and Chronic Health Evaluation II (APACHE II) score, Sequential Organ Failure Assessment (SOFA) *score*, comorbidities, days in the hospital before ICU admission, and C-reactive protein (CRP) (Appendix 1). We used the variant of the NUTRIC score that replaces interleukin-6 (IL-6) for CRP, since it uses an inflammatory biomarker and has been validated in our population.^(17)^ NUTRIC was considered to indicate high nutritional risk if the score was ≥ 6 points and low if it was ≤ 5 points. NUTRIC was calculated by the medical staff.

For the assessment of PHC, a 24-hour urine sample was collected, in which the urea level was determined and TUNes was calculated from the total urinary urea (TUU) using the following formula: $\mathrm{TUNes}=\left[\left(\mathrm{TUU}/2\right)\;x\;1.10\right]+2.35$.^(11)^ Protein hypercatabolism was classified as follows: absent (TUNes < 5g/day); mild (TUNes 5 - 10g/day), moderate (TUNes 10 - 15g/day), and severe (TUNes > 15g/day).^(11)^

Acute kidney failure was defined as serum creatinine > 1.2mg% and/or glomerular filtration < 50mL/min at any time during the observation period. For this study and due to the lack of consensus about the cutoff points to establish a diagnosis of renal insufficiency from urinary nitrogen, it was established arbitrarily.^(7-11)^

For the measurement of CRP in mg/dL, the particle-enhanced immunoturbidimetric method (Roche Diagnostics GMBH^®^) was used. The urinalysis was performed with the enzymatic method (Kinetic test with urease and glutamate dehydrogenase - Cabas 6000®) to determine urinary urea and the colorimetric kinetic method (2nd generation Creatinine Jaffe® - Cabas 6000®) to determine urinary and serum creatinine.

The SATI-Q software was used as a data recording instrument and for the automatic calculation of the APACHE II, SOFA, Simplified Acute Physiology Score II (SAPS II) scores. SATI-Q is a computer tool used to record data referring to quality standards, sponsored by the Argentine Society of Intensive Therapy (*Sociedad Argentina de Terapia Intensiva* - SATI). Data loading was performed in real time by properly trained medical and nursing personnel.

The first day spanned from the time of admission to the ICU until the assessment at 8 a.m. the following day, so this period could be less than 24 hours. The second day and subsequent days ran from 8 a.m. to 8 a.m. the following day. Urine samples were collected from patients enrolled in the study on the second day (T1) and the fourth day (T2) of admission to the ICU. At both times, an isolated urine sample was collected at 8 a.m., in which the U/CI was calculated, followed by a 24-hour urine sample, in which the urea level was analyzed and the TUN was estimated. The measurement on the fourth day was established based on the recommendations of the A.S.P.E.N.-SCCM guide so that we could adopt a nutritional strategy at that time according to the nutritional risk obtained at admission.^(3)^ The calories and proteins provided by the nutritional support at these times were recorded.

### Statistical analysis

The categorical variables are summarized as the number of cases and percentages, and quantitative variables are summarized as the mean ± standard deviation, or as the median (interquartile range) in the presence of asymmetry. To evaluate the comparative hypotheses, the proportions test, chi-squared test, or Fisher’s exact test was used in the case of small samples, and the two-tailed t-test was used for independent samples and the Mood test for medium-sized samples. To compare the means of quantitative variables between consecutive measurement times, the paired t-test was used. In all tests, the significance level adopted was 0.05. The degree of association between the quantitative variables was evaluated with Pearson and Spearman correlation coefficients and their significance with the normal z-test. To determine the capacity of the U/CI, APACHE II score, and NUTRIC score to diagnose sPHC on the fourth day (T2), binary logistic regression was used for each of the indicators separately. Specificity, sensitivity, positive and negative predictive values, positive and negative likelihood ratios, and areas under the Receiver Operating Characteristic (ROC) curves (AUCs) were estimated specifically and via confidence intervals as measures of efficiency. Minitab 18 software was used for data processing.

The study was approved by the Teaching and Research Committee of the *Hospital Escuela Eva Perón*. To protect the confidentiality of patients, the first and last name was replaced by an alphanumeric code. This information was only used by the authors and was never available to people outside the study.

## RESULTS

**Figure 1:**
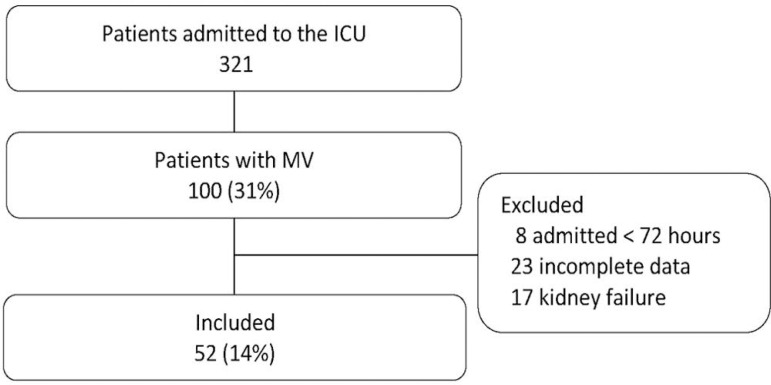
Flow chart of patient selection. ICU - intensive care unit, MV - mechanical ventilation.

In the 6-month study period, 321 patients were admitted to the ICU of the *Hospital Escuela Eva Perón*, of whom 52 were analyzed in this study (Figure 1). The mean age was 41.73 years (± 16.76), and 67.31% of them were male. The most frequent admission pathology was trauma (40.4%), followed by neurological (21.15%) and sepsis pathologies (13.46%). The mean APACHE II, SAPS II, and SOFA scores were 16.00 (± 6.88), 37.54 (± 15.05), and 5.98 (± 3.27), respectively. The mean score on the NUTRIC scale was 2.59 (± 1.71) points. Eighty-four percent of patients were classified as having low nutritional risk by the NUTRIC scale. There were no contributions of enteral feeding at T1 or T2, and the average calorie and protein intakes were 853 kcal and 32 g, respectively. The mean hospitalization was 13.12 (± 13.28) days, the time on AMV was 9.90 (± 11.90) days, and the mortality in the ICU was 30.77%.

The average TUNes on the second day and fourth day was 12.92g (± 4.60) and 16.20 (± 6.49) (p = 0.000), respectively. The average U/CI was 15.98 (± 7.63) and 19.60 (± 10.36) (p = 0.012).

Severe protein hypercatabolism (TUNes > 15g) was found in 14 (26.9%) patients on the second day of hospitalization and in 29 (55.7%) on the fourth day. Comparisons on the second day and fourth day of patients with or without sPHC can be observed in table 1.

**Table 1:** Comparisons between groups with and without severe protein hypercatabolism in T1 and T2

Variables	T1			T2		
	sPHC (n = 14)	No sPHC (n = 38)	p value	sPHC (n = 29)	No sPHC (n = 23)	p value
Age (years)	43.5 ± 16.3	41.1 ± 17.1	0.649	43.6 ± 18.7	39.3 ± 13.9	0.366
Male sex	12 (85.7)	23 (60.52)	0.107	18 (62.0)	17 (73.9)	0.393
Reasons for admission						
Medical pathology	7 (50.00)	16 (42.10)		12 (41.37)	11 (47.82)	
Multiple injuries	1 (7.14)	6 (15.78)	0.674	14 (48.27)	8 (34.72)	0.563
Surgery	6 (42.85)	16 (42.10)		3 (10.34)	4 (17.39)	
Pathologies						
Trauma	6 (42.85)	15 (39.47)		13 (44.82)	8 (34.78)	
Neurological	2 (14.28)	9 (23.68)		3 (10.34)	8 (34.78)	
Sepsis	3 (21.42)	4 (10.52)	----	5 (17.24)	2 (8.69)	0.073
Postoperative	0 (0)	4 (10.52)		3 (10.34)	1 (4.34)	
Respiratory	1 (7.14)	2 (5.26)		3 (10.34)	0 (0)	
Other	2 (14.28)	4 (10.52)		2 (6.89)	4 (17.39)	
APACHE II	18.57 ± 5.77	15.05 ± 7.09	0.103	17.83 ± 6.22	13.70 ± 7.12	0.030
SAPS II	44.6 ± 14.5	34.9 ± 14.6	0.040	41.3 ± 13.9	32.7 ± 15.4	0.039
SOFA	6.64 ± 2.34	5.74 ± 3.55	0.381	6.24 ± 2.81	5.65 ± 3.82	0.525
NUTRIC	3.00 ± 1.52	2.74 ± 1.93	0.647	3.24 ± 1.84	2.26 ± 1.66	0.052
CRP mg %	3.00 (9.18)	1.40 (9.78)	0.532	13.10 (22.7)	13.30 (11.0)	0.780
TUN g/24 hours	18.93 ± 2.85	10.71 ± 2.76	0.000	20.44 ± 5.31	10.86 ± 2.88	0.000
U/CI	18.21 (11.29)	14.30(11.01)	0.211	20.82 (10.4)	13.32 (7.83)	0.051
Median ICU	9.50 (5.25)	11.00 (15.00)	0.044	10.00 (12.50)	10.00 (10.0)	0.642
Median MV	5.00 (6.25)	7.50 (12.50)	0.087	6.00 (6.50)	9.00 (8.00)	0.100
Deceased	5 (35.71)	11 (28.94)	0.738	7 (24.13)	9 (39.13)	0.365

sPHC - severe protein hypercatabolism; APACHE II - Acute Physiology and Chronic Health Evaluation II; SAPS II - Simplified Acute Physiology Score II; SOFA - Sequential Organ Failure Assessment; NUTRIC - Nutrition Risk in the Critically Ill; CRP - C-reactive protein; TUN - total urinary nitrogen; U/CI - urea/creatinine index; ICU - intensive care unit; MV - mechanical ventilation. Results expressed as mean ± standard deviation, n (%) or median (interquartile range).

**Figure 2:**
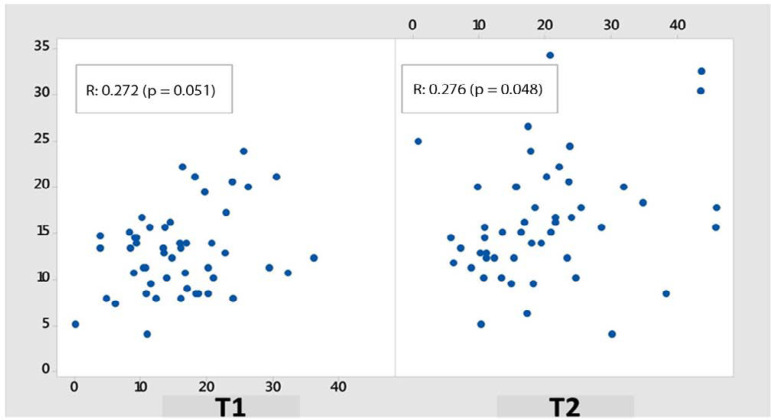
Relationship between total urinary nitrogen and urea/creatinine index on T1 and T2.

On the second day, the Pearson and Spearman correlations between TUNes and U/CI were 0.272 (p = 0.051) and 0.161 (p = 0.255), respectively, and on the fourth day, they were 0.276 (p = 0.048) and 0.297 (p = 0.032) (Figure 2).

**Figure 3:**
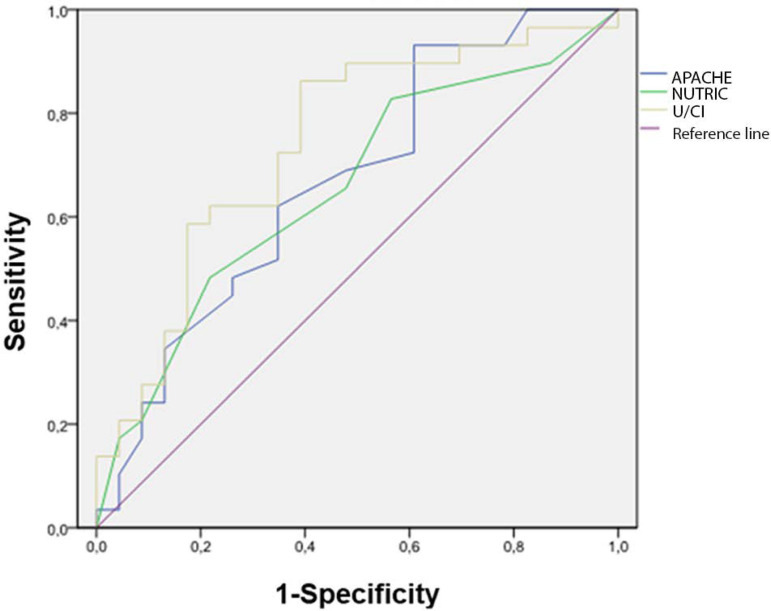
Receiver Operating Characteristic curves for the diagnosis of severe protein hypercatabolism at T2 using the urea/creatinine index, Acute Physiology and Chronic Health Evaluation II, and Nutrition Risk in the Critically Il. APACHE II - Acute Physiology and Chronic Health Evaluation II; NUTRIC - Nutrition Risk in the Critically Ill; U/CI - urea/creatinine index

Receiver Operating Characteristic curves were constructed using the binary logistic model to predict sPHC on the fourth day according to each indicator: U/CI, APACHE II, and NUTRIC (Figure 3). The calculated AUCs were 0.741 (95% confidence interval - 95%CI 0.602 - 0.880), 0.669 (95%CI 0.519 - 0.818), and 0.656 (95%CI 0.506 - 0.806), respectively.

Table 2 shows the efficiency measures of U/CI on the fourth day, with a cutoff value of 16.15, for the diagnosis of sPHC.

**Table 2:** Efficiency measures of the urea/creatinine index for diagnosing severe protein hypercatabolism at T2

Indicator		95%CI
Sensitivity %	79.31	59.74 - 91.29
Specificity %	60.87	38.78 - 79.53
Predictive value + %	71.88	53.02 - 85.60
Predictive value - %	70.00	45.67 - 87.16
Likelihood ratio + (LR+)	2.03	1.18 - 3.49
Likelihood ratio - (LR-)	0.34	0.16 - 0.74

95%CI - 95% confidence interval; LR - likelihood ratio.

## DISCUSSION

The most notable finding of our study is that the U/CI in isolated urine had a certain ability to estimate the PHC of the critically ill patient but cannot replace TUN for its assessment.

Swaminathan et al. studied the correction of nitrogen excretion by body size and found a close correlation between U/CI in 24-hour urine and U/CI in isolated urine, even in the group of critically ill patients.^(13)^ The study was conducted in the late 1970s, and the relationship was based on isolated morning urine.

On the other hand, the work of García Arévalo et al. did not clarify the day of hospitalization, the time at which the isolated urine sample was taken for measuring U/CI, or the time at which the 24-hour urine sample was taken.^(14)^ Although they explored Swaminathan’s suggestion of using U/CI to determine 24-hour nitrogen excretion, they used several formulas in the search for greater precision, which reduced the bedside applicability and was detrimental to the dynamics that is needed from such an estimator.^(15)^

Mountokalakis et al. used only U/CI in urine samples isolated from mid-morning to estimate the protein catabolic slope in 29 patients with stroke and 18 elective surgical patients but did not compare it with 24-hour urine nitrogen results.^(16)^

The fact that our study analyzed second- and fourth-day TUNes and U/CI in isolated urine before collecting the 24-hour urine sample allowed us to overcome the limitations of previous studies and contemplate their results in the context of the current guidelines of A.S.P.E.N. - SCCM on choosing the intensity of nutritional therapy based on nutritional risk and on the phase (*ebb/flow*) of the patient’s critical disease.^(3)^ In this sense, although the high frequency of sPHC in the first days of evolution of the critical illness (27% and 55% on the second and fourth days) of our patients is typical and reflects the metabolic stress response, 84% of our patients were considered to have low nutritional risk by NUTRIC. The average values of TUNes at T1 are similar to those reported by Arabi et al. in a general ICU population, whose values in the low- and high-risk groups categorized by NUTRIC were 11.5 and 10.4g under permissive feeding and 12g and 9.5g under standard feeding, respectively.^(18)^ On the other hand, in a trauma population, Dickerson et al. found a greater nitrogen excretion, close to 20 g, around the fourth day.^(19)^

The significant increase in the average value of TUNes between the second and fourth day was mirrored by U/CI. However, the U/CI in urine isolated before the 24-hour urine loses its discriminative capacity by the second day, and by the fourth day it only has a marginal association, at the limit of statistical significance, with the sPHC. It has a better correlation with TUNes in the day-4 sample, although discrete and at the expense of a large spread. The determinants of urinary nitrogen excretion vary over time during the acute phase of critical illness. The protein intake that came from nutritional therapy may have contributed to the urinary nitrogen excretion, but considering the endogenous catabolism index suggested by Bistrian et al.^(20)^ and that the enteral route was used in continuous infusion with a low nutritional adequacy at the time of the study, it could not have significantly influenced the results. On the other hand, in critically ill patients, dynamic changes occur in both basal metabolism (hyper-/hypothermia, hyper-/hypovolemia, sensory motor arousal, muscle relaxants, mechanical ventilatory assistance etc.) and kidney function (hyperfiltrating kidney syndrome in young polytraumatized patients, decreased filtration in patients with acute kidney failure), which could explain the discrete correlation between the values of the isolated samples and the 24-hour sample. The data obtained in our cohort, which had an average age of 40 years, a prevalence of traumatic pathology, and an exclusion criterion of renal insufficiency (defined arbitrarily), support the fact that an index of isolated urine (U/CI) does not replace 24-hour urine collection and the subsequent estimation of the TUN for the determination of the PHC.

On the other hand, if it is recognized that critically ill patients behave as a heterogeneous population in terms of nutritional risk and that not all will respond in the same way to nutritional interventions, the determination of sPHC could complement the recommended nutritional risk scales (NUTRIC or NRS 2002) and could be useful to achieve a greater benefit from nutritional therapy by stimulating a behavior aimed at providing proteins early and according to the intensity of the protein loss.^(1,21-23)^

Currently, there is no simple and dynamic tool to screen for sPHC that would help to select the patients in whom to perform a 24-hour urine collection to estimate the TUN. In this sense, in our cohort, the U/CI before 24-hour urine collection on the fourth day showed better discrimination of sPHC than the disease severity score APACHE II and the nutritional risk score NUTRIC, although the curves did not have significant differences (AUC 0.741, 0.669, and 0.656, respectively). The U/CI with a cutoff of 16.15 detected 23 of 29 (79.31%) of patients with severe PHC and ruled out the presence of it in 14 of 23 (60.87%) patients who did not present it. Its usefulness should be addressed in depth in future studies.

A main limitation of our study is that it is a follow-up analysis of a small sample. The fact that there was no significant difference in mortality in patients with sPHC may be due to the nature of the study. A prospective study, with a greater number of patients and the participation of several centers, would be necessary to evaluate a possible effect of sample size on both the mortality of the sPHC and the discriminative power of the U/CI. The exclusion of patients with kidney failure who did not require mechanical ventilation from first admission precludes the extrapolation of our results to these populations, limiting their external validity in the real ICU setting. The TUN was not validated by direct measurement (Kjeldahl or pyrogen chemiluminescence)

## CONCLUSION

The urea/creatinine index measured on the fourth day has a certain ability to estimate severe protein hypercatabolism (as defined by total urinary nitrogen) but does not replace total urinary nitrogen in critically ventilated patients without kidney failure. Due to its reasonable sensitivity, it could be used as a screening criterion to identify which patients to take a 24-hour urine sample from.

## Appendix 1

**Table:** Variant of the Nutrition Risk in the Critically Ill Scale with C-reactive protein

NUTRIC scale				
Variables	Points			
	0	1	2	3
Age (years)	≤ 49	50 - 74	≥ 75	
APACHE II (points)	≤ 14	15 - 19	20 - 28	≥ 29
SOFA (points)	≤ 5	6 - 9	≥ 10	
Comorbidities	≤ 1	≥ 2		
Days before ICU admission	0	≥ 1		
CRP	< 10	≥ 10		
**Bajo riesgo**	**0 - 5**	**High risk**		**6 - 10**

NUTRIC - Nutrition Risk in the Critically Ill; APACHE II - Acute Physiology and Chronic Health Evaluation II; SOFA - Sequential Organ Failure Assessment; ICU - intensive care unit; CRP - C-reactive protein.

Source: Moretti D, Bagilet DH, Buncuga M, Settecase CJ, Quaglino MB, Quintana R. [Study of two variants of nutritional risk score "NUTRIC" in ventilated critical patients]. Nutr Hosp. 2014;29(1):166-72. Spanish.^(17)^
